# Differential modulation and prognostic values of immune-escape genes in uveal melanoma

**DOI:** 10.1371/journal.pone.0210276

**Published:** 2019-01-17

**Authors:** Maria Sofia Basile, Emanuela Mazzon, Andrea Russo, Santa Mammana, Antonio Longo, Vincenza Bonfiglio, Matteo Fallico, Rosario Caltabiano, Paolo Fagone, Ferdinando Nicoletti, Teresio Avitabile, Michele Reibaldi

**Affiliations:** 1 Department of Biomedical and Biotechnological Sciences, University of Catania, Catania, Italy; 2 IRCCS Centro Neurolesi Bonino Pulejo, C.da Casazza, Messina, Italy; 3 Department of Ophthalmology, University of Catania, Catania, Italy; 4 Department 'G.F. Ingrassia', Section of Anatomic Pathology, University of Catania, Catania, Italy; Universidade de Sao Paulo, BRAZIL

## Abstract

Uveal melanoma (UM) is the most common primary intraocular cancer in adults. In the present study, we aimed to characterize the immunological features of primary UM cancer and to provide an association with prognostic markers and outcome. Also, we assessed the influence of the microenvironment on the expression of inhibitory immune checkpoints in UM. Genes of interest included MHC Class I and Class II molecules, as well as inhibitory immune-checkpoints, i.e. PDL1, PDL2, B7-H3, B7-H4, TBFRSF6B, CD47, CD155, GAL9, HVEM and CD200. We observed significant lower levels of MHC genes in UM cells as compared to normal uveal melanocytes. Unexpectedly however, the expression levels of most of the analyzed inhibitory immune-checkpoint genes were not different in cancer cells as compared to normal melanocytes, with the exception of CD200 and HVEM, that resulted significantly reduced. On the other hand, PDL1 inversely correlated with OS, PFS and thickness of the tumor. Also, PDL1, along with PDL2, expression significantly increased under inflammatory conditions. Finally, for the first time, we propose a possible role for CD47 in the immune evasive properties of UM. We show here that CD47 is significantly upregulated by UM cells following inflammatory stimuli and that it represents a good independent predictor of disease progression. The results from this study may propel advances in the development of immune-based therapies for UM patients.

## Introduction

Uveal melanoma (UM) is the most common primary intraocular cancer in adults [1,2]. The 5-year survival rate is between 50% and 70% and up to 50% of patients develop metastases within 36 months with a median survival of 6 months after metastasis occurrence [1,2]. Unfavorable prognostic markers are the presence of epithelioid cells, large tumor diameter, anterior localization and chromosome 3 monosomy. Also, on the contrary to other cancers, the presence of tumor-infiltrating lymphocytes is associated to a poor outcome [3–5].

Recently, UMs have been classified as either Class 1 (with low metastatic risk) or Class 2 (with high metastatic risk), based on the transcriptomic profile of 12 genes. Class 1 UMs are further divided into Class 1a and Class 1b, the latter bearing a worse prognosis. Furthermore, the preferentially expressed antigen in melanoma (PRAME) gene has been reported to be an independent prognostic biomarker [6]. The liver accounts for 80–91% of the metastases. Although both cutaneous melanomas and UM originate from the melanocyte, their clinical behavior and underlying molecular mechanisms differ significantly [7]. For example, unlike cutaneous melanoma where metastasis to the central nervous system (CNS) occur in 40–60% of cases, only 4–15% of UM metastasize to CNS. The reasons for this discrepant metastatic pattern have so far not been dismantled.

The better understanding of biological differences and gene signatures between cutaneous and UM are clearly important to identify the factors that confer lower CNS metastatic potential to uveal vs. cutaneous melanoma.

Standard of care treatment of UM is represented by local radiation therapy, i.e. brachytherapy or charged-particle and proton-beam radiation, and enucleation for very large tumors [1,2]. For metastatic UM, no FDA-approved standard of care is available [6]. While the development of immune checkpoint inhibitors, targeting cytotoxic T-lymphocyte-associated antigen 4 (CTLA-4) and programmed cell death-1 (PD-1), have dramatically increased the outcome for patients with advanced cutaneous melanoma, a comparable clinical benefit has not been observed for unresectable/metastatic UM. This can be partly explained by the observation that TILs from cutaneous melanoma have higher anti-tumor reactivity as compared to those from UM samples [8]. It is believed that since the eye is an immune privileged site, the tumor and its metastases show local immune-suppressive and immune-evasive features that affect the efficacy of current immunotherapies [9–12].

In the present study, we aimed to characterize the immunological features of primary UM cancer and to provide an association with prognostic markers and outcome. Also, we assessed the influence of the microenvironment on the expression of inhibitory immune checkpoints in UM. The results from this study may propel advances in the development of immune-based therapies for primary and recurrent UM cases.

## Materials and methods

### Immunological profiling of uveal melanoma

In order to evaluate the immunological differences between UM and normal uveal melanocytes, the publicly available GSE62075 dataset was obtained from Gene Expression Omnibus (GEO, https://www.ncbi.nlm.nih.gov/geo/). GSE62075 included gene expression profiling of three primary cultures of human uveal melanocytes and of three human UM cell lines, the T115, T142 and T143 cells, at low culture passages (between 2 and 7). The G4851A SurePrint G3 Human GE 8x60 K array slide platform was used. Detailed description of the experimental design and procedures can be retrieved from the relative publication [13]. Data were analyzed using LIMMA (Linear Models for Microarray Analysis) and genes with a Benjamini & Hochberg false discovery rate<0.05 were considered to be significant.

### Immunological changes in uveal melanoma under inflammatory conditions

To determine whether pro-inflammatory factors from activated T cells altered the immunological profiling of UM, the GSE55983 microarray dataset was used. GSE55983 included data from the three primary human UM cell lines, Mel290, Mel270 and 92.1. CD3+ T cells were isolated from whole blood of healthy donors, activated using anti-CD3/CD28 antibodies and cultured in 0.4 μm membrane inserts over UM cell lines, for 64 hours. After the incubation period, total RNA was collected from the UM cell lines and genome-wide transcriptomic expression was obtained using the Affymetrix Human Gene 1.0 ST microarray platform [14].

### Identification of clinical predictors

Genes of interest included MHC Class I and Class II molecules, as well as inhibitory immune-checkpoints, i.e. PDL1, PDL2, B7-H3, B7-H4, TBFRSF6B, CD47, CD155, GAL9, HVEM and CD200 [15]. In order to evaluate whether the expression levels of inhibitory immune-checkpoints and HLA genes in UM are able to predict prognosis or are associated to relevant clinical features, RNA Seq data from the TCGA datasets were downloaded through the cBioportal web-based utility (http://www.cbioportal.org; http://bit.ly/2HAJyjG). Data from 80 primary tumors, with no neoadjuvant therapy prior to excision were selected. Clinical data included metastasis free survival, overall survival, tumor diameters, BAP1 mutations and mitotic count.

### Statistical analysis

Gene expression differences between normal uveal melanocytes and UM were evaluated using unpaired two-tailed Student’s t test. Enrichment of the “Immune Response” Biological Process (GO:0006955) among the significantly modulated genes in GSE62075 was calculated using an hypergeometric test. Paired two-tailed Student’s t test was used to assess differences in gene expression in UM cells following pro-inflammatory stimulation. Statistical correlation between two continuous variables was calculated by partial correlation accounting for disease status, histological type, BAP1 mutation and adjuvant therapy. For each gene, predicted values were obtained by Multivariate General Linear Model using BAP1, Histological type and Adjuvant therapy as Fixed Factors and Mitotic count, Basal Diameter and Thickness as Covariates. Receiver Operating Characteristic (ROC) curves were constructed using disease status (recurrent disease) as nominal variable. Survival analysis was performed using Kaplan-Meier and its significance analyzed by log-rank (Mantel-Cox) test. Continuous variables were dichotomized using median value as cut off. For all the analysis, a p-value <0.05 was considered statistical significant. Statistical analysis was performed with GraphPad Prism 5 (GraphPad Software, San Diego, C, US) and SPSS 24 (IBM SPSS Statistics, IBM Corporation, Armonk, NY, US).

## Results

### Modulation of immune-related molecules in uveal melanoma

**Fig 1:**
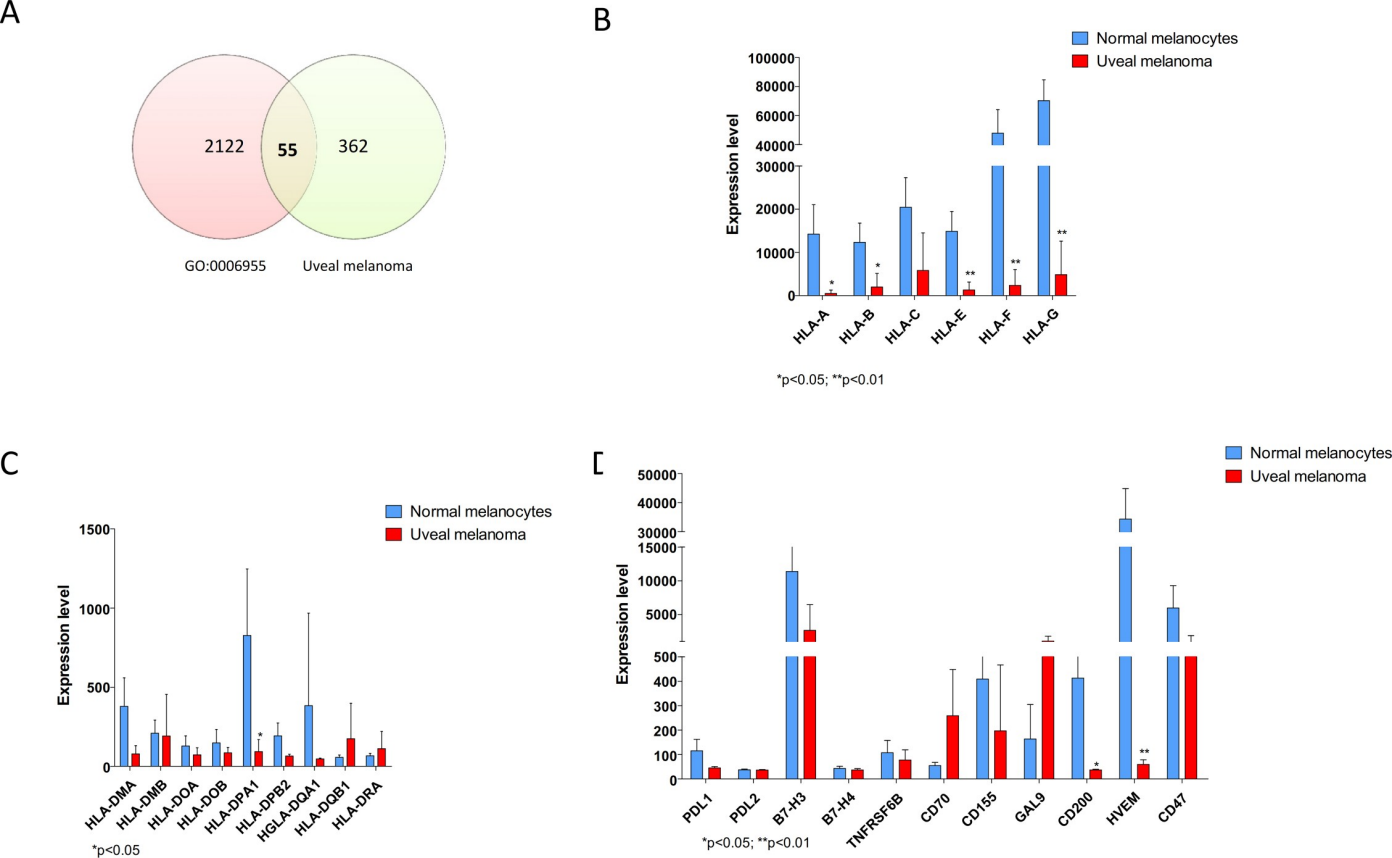
Immunological profiling of uveal melanoma. A. Venn diagram showing the number of significantly modulated genes in GSE62075 overlapping the “Immune response” genes (GO:0006955); B. MHC class I gene expression in uveal melanoma as determined in GSE62075; C. MHC class II gene expression in uveal melanoma as determined in GSE62075; D. Immune checkpoint gene expression in uveal melanoma as determined in GSE62075.

Analysis of GSE62075 revealed that in UM, 417 genes are significantly modulated as compared to normal uveal melanocytes. Fifty-five genes resulted in common with genes belonging to the “Immune Response” Biological Process (GO:0006955), reaching a strong statistical significance (p<0.001) (Fig 1A). Next, we wanted to compare the expression of MHC Class I and Class II molecules, as well as inhibitory immune-checkpoints, i.e. PDL1, PDL2, B7-H3, B7-H4, TBFRSF6B, CD47, CD155, GAL9, HVEM and CD200, in UM in comparison to normal uveal melanocytes. MHC class I molecules resulted significantly downregulated in UM as compared to normal uveal melanocytes (Fig 1B), while no differences were observed for the MHC Class II genes, with the exception for HLA-DPA1 (Fig 1C). As regards the immune checkpoints investigated, no modulation was observed for PDL1, PDL2, B7-H3, B7-H4, TBFRSF6B, CD47, CD155, and GAL9 (Fig 1D). However, HVEM and CD200 were significantly downregulated in UM (p = 0.042 and p = 0.005).

### Inflammation drives immune escape in uveal melanoma

**Fig 2:**
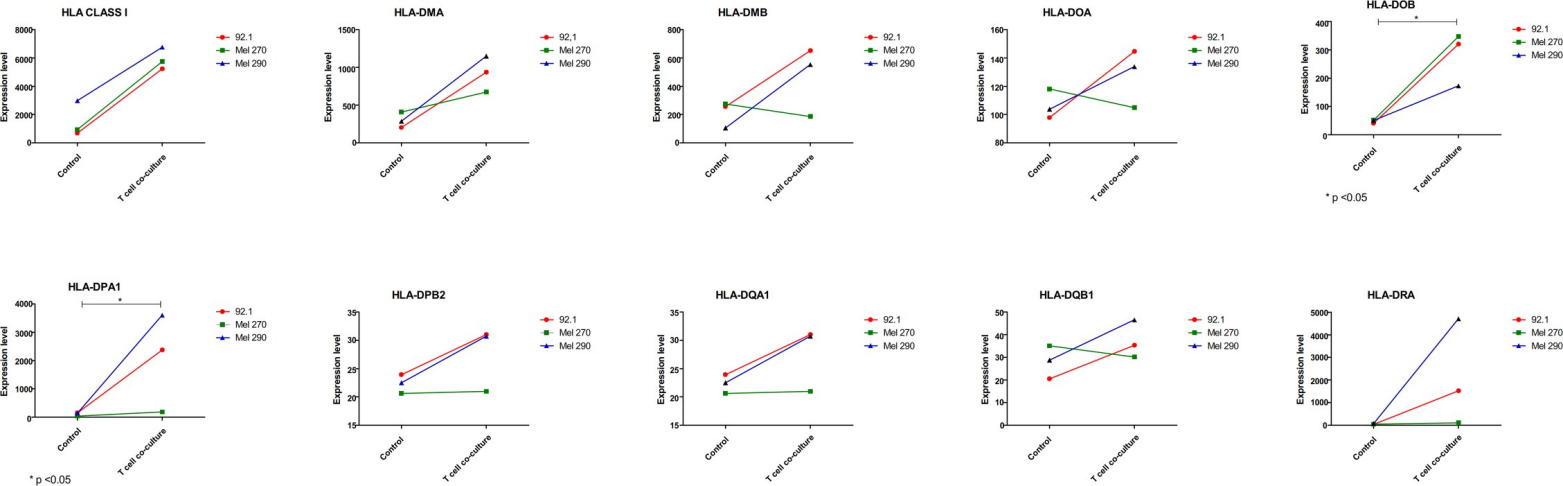
Inflammation drives immune escape in uveal melanoma. GSE55983 included data from the three primary human uveal melanoma cell lines, Mel290, Mel270 and 92.1. CD3+ T cells were isolated from whole blood from healthy donors, activated using anti-CD3/CD28 antibodies and cultured in 0.4 μm membrane inserts over uveal melanoma cell lines, for 64 hours. The expression levels of MHC Class I and II genes have been determined.

**Fig 3:**
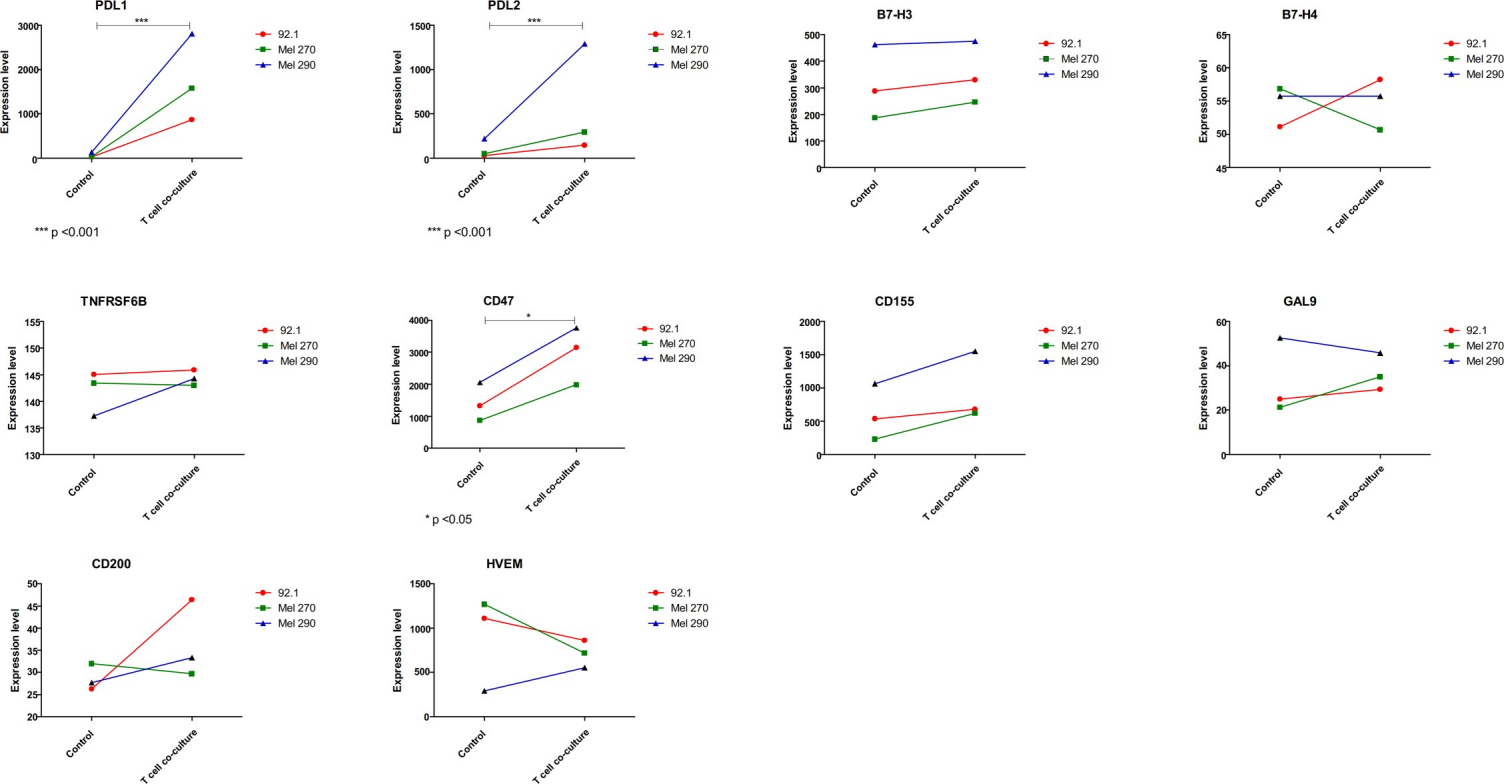
Inflammation drives immune escape in uveal melanoma. GSE55983 included data from the three primary human uveal melanoma cell lines, Mel290, Mel270 and 92.1. CD3+ T cells were isolated from whole blood from healthy donors, activated using anti-CD3/CD28 antibodies and cultured in 0.4 μm membrane inserts over uveal melanoma cell lines, for 64 hours. The expression levels of immune-checkpoint genes have been determined.

The GSE55983 microarray dataset was used to assess the modulation of the genes of interest upon inflammatory conditions. A trend of increase (p = 0.052) was observed for the MHC Class I molecules (Fig 2). Also, a significant upregulation in HLA-DOB and HLA-DPA1 was found (p<0.05). A significant modulation was observed in PDL1 and PDL2 gene expression levels (p<0.001), that were dramatically increased after incubation with activated T cell supernatant, as well as in CD47 levels (p<0.05) (Fig 3). It is likely that the increase in expression of the above-mentioned genes are attributable to the effect of IFN-gamma secreted by activated T cells, as exposure of 92.1 cells to IFN-gamma (200 ng/ml) was associated to an upregulation of PDL-1, HLA-A, HLA-DOB and CD47 (S1 Fig).

To evaluate the association of the genes of interest with patients’ and tumor characteristics, a partial correlation was performed to account for disease status, histological type and adjuvant therapy. As expected, OS and progression free survival (PFS) resulted significantly correlated (Table 1). Also, a trend of correlation was observed between mitotic count and tumor diameters (Table 1). PDL1 inversely correlated with OS, PFS and thickness of the tumor. Similarly, a significant inverse correlation was observed between HLA-A, HLA-B, HLA-H HLA-DPA and PFS (Table 1). No assessment could be performed for B7-H4, whose expression levels were below detection sensitivity. A significant correlation was observed in the expression of all the Class I and Class II MHC molecules, as well as for MHC genes and PDL1 and PDL2 (Table 2). Moreover, GAL9 significantly correlated with MHC class I and PDL1 expression (Table 2). Finally, a significant correlation (p = 0.001) was observed between PDL1 and CD47 expression levels (Table 2).

**Table 1:** Correlation between gene levels and clinical variables as determined in the uveal melanoma samples from the TGCA cohort of patients. Partial correlation was performed to account for clinical confounding variables.

Control Variables			OS	PFS	MitoticCount	BasalDiameter	Thickness	HLAA	HLAB	HLAC	HLAF	HLAG	HLAH	HLADMA	HLADMB	HLADOA	HLADOB	HLADPA	HLADQA1	HLADQB1	HLADRA	PDL1	PDL2	B7H3	TNFRSF6B	GAL9	CD47	CD200	HVEM	CD155
DiseaseStatus & HistoType & Adjuvant & BAP1MUT	OS	Correlation	1.000	0.983	0.014	0.127	0.243	-0.494	-0.352	-0.347	-0.239	-0.226	-0.458	-0.278	-0.291	-0.289	-0.192	-0.312	-0.309	-0.284	-0.281	-0.376	-0.170	0.038	-0.047	-0.107	-0.101	0.232	-0.105	0.112
		Significance (2-tailed)		**0.000**	0.935	0.441	0.136	**0.001**	**0.028**	**0.030**	0.143	0.166	**0.003**	0.086	0.072	0.074	0.241	0.053	0.056	0.080	0.083	**0.018**	0.301	0.820	0.776	0.518	0.540	0.155	0.523	0.498
	PFS	Correlation	0.983	1.000	-0.006	0.123	0.254	-0.484	-0.364	-0.373	-0.254	-0.219	-0.453	-0.286	-0.306	-0.313	-0.192	-0.322	-0.301	-0.280	-0.294	-0.395	-0.171	0.040	-0.019	-0.111	-0.162	0.195	-0.119	0.092
		Significance (2-tailed)	**0.000**		0.973	0.456	0.119	**0.002**	**0.023**	**0.019**	0.119	0.181	**0.004**	0.077	0.059	0.053	0.243	**0.046**	0.063	0.084	0.069	**0.013**	0.297	0.808	0.907	0.501	0.323	0.235	0.470	0.578
	MitoticCount	Correlation	0.014	-0.006	1.000	0.287	0.292	-0.031	-0.036	-0.081	-0.107	0.057	0.038	0.037	-0.084	-0.111	-0.087	-0.027	0.069	0.015	-0.067	-0.196	-0.183	-0.036	-0.181	-0.014	-0.069	-0.107	-0.006	0.253
		Significance (2-tailed)	0.935	0.973		0.077	0.071	0.852	0.830	0.622	0.517	0.731	0.817	0.821	0.611	0.503	0.597	0.872	0.675	0.930	0.685	0.231	0.266	0.827	0.270	0.934	0.677	0.518	0.972	0.120
	BasalDiameter	Correlation	0.127	0.123	0.287	1.000	0.512	-0.127	-0.026	-0.149	-0.027	-0.055	-0.073	0.028	-0.034	-0.084	-0.025	-0.081	-0.015	-0.013	-0.047	-0.148	-0.068	0.024	0.225	0.171	-0.110	0.004	-0.090	0.316
		Significance (2-tailed)	0.441	0.456	0.077		**0.001**	0.440	0.874	0.365	0.868	0.740	0.658	0.866	0.840	0.612	0.880	0.622	0.926	0.938	0.775	0.369	0.682	0.886	0.168	0.298	0.505	0.979	0.587	0.050
	Thickness	Correlation	0.243	0.254	0.292	0.512	1.000	-0.137	-0.062	-0.114	-0.154	-0.153	-0.001	-0.074	-0.148	-0.187	-0.185	-0.120	-0.075	-0.124	-0.149	-0.367	-0.298	-0.089	0.078	0.184	-0.164	0.025	-0.047	0.204
		Significance (2-tailed)	0.136	0.119	0.071	**0.001**		0.406	0.706	0.490	0.350	0.351	0.997	0.653	0.369	0.254	0.259	0.467	0.650	0.452	0.364	**0.021**	0.065	0.590	0.635	0.263	0.317	0.880	0.777	0.213

**Table 2:** Correlation between gene expression levels as determined in the uveal melanoma samples from the TGCA cohort of patients. The Correlation Index (R^2^) is reported.

	HLAA	HLAB	HLAC	HLAF	HLAG	HLAH	HLADMA	HLADMB	HLADOA	HLADOB	HLADPA	HLADQA1	HLADQB1	HLADRA	PDL1	PDL2	B7H3	TNFRSF6B	GAL9	CD47	CD200	HVEM	CD155
HLAA	1	0.81	0.807	0.685	0.286	0.962	0.717	0.651	0.717	0.468	0.755	0.67	0.603	0.687	0.572	0.51	0.284	0.1	0.317	-0.041	-0.156	0.368	-0.099
HLAB	0.81	1	0.899	0.919	0.252	0.824	0.816	0.848	0.892	0.735	0.862	0.814	0.793	0.882	0.748	0.752	0.015	0.262	0.411	0.246	-0.119	0.133	-0.058
HLAC	0.807	0.899	1	0.793	0.272	0.837	0.713	0.724	0.841	0.523	0.783	0.625	0.616	0.762	0.673	0.593	0.032	0.232	0.396	0.208	0.001	0.233	-0.099
HLAF	0.685	0.919	0.793	1	0.195	0.665	0.84	0.936	0.933	0.909	0.891	0.845	0.892	0.962	0.701	0.887	-0.026	0.211	0.24	0.302	-0.051	0.044	-0.005
HLAG	0.286	0.252	0.272	0.195	1	0.336	0.089	0.107	0.116	0.07	0.132	0.176	0.079	0.12	0.349	0.119	0.024	0.114	0.452	-0.06	0.065	0.019	0.3
HLAH	0.962	0.824	0.837	0.665	0.336	1	0.723	0.621	0.704	0.418	0.745	0.655	0.573	0.665	0.562	0.447	0.283	0.124	0.424	-0.041	-0.157	0.375	-0.068
HLADMA	0.717	0.816	0.713	0.84	0.089	0.723	1	0.916	0.907	0.77	0.936	0.908	0.902	0.921	0.561	0.759	0.289	-0.068	0.19	0.004	-0.267	0.262	0.006
HLADMB	0.651	0.848	0.724	0.936	0.107	0.621	0.916	1	0.96	0.893	0.952	0.925	0.954	0.988	0.659	0.875	0.018	0.045	0.123	0.227	-0.13	0.084	-0.028
HLADOA	0.717	0.892	0.841	0.933	0.116	0.704	0.907	0.96	1	0.803	0.957	0.859	0.884	0.973	0.697	0.814	0.053	0.027	0.142	0.234	-0.112	0.168	-0.074
HLADOB	0.468	0.735	0.523	0.909	0.07	0.418	0.77	0.893	0.803	1	0.781	0.828	0.915	0.896	0.567	0.918	-0.035	0.121	0.083	0.29	-0.129	-0.099	0.022
HLADPA	0.755	0.862	0.783	0.891	0.132	0.745	0.936	0.952	0.957	0.781	1	0.926	0.926	0.966	0.627	0.787	0.123	-0.003	0.175	0.112	-0.174	0.218	-0.066
HLADQA1	0.67	0.814	0.625	0.845	0.176	0.655	0.908	0.925	0.859	0.828	0.926	1	0.963	0.92	0.625	0.812	0.101	-0.03	0.174	0.102	-0.218	0.085	0.025
HLADQB1	0.603	0.793	0.616	0.892	0.079	0.573	0.902	0.954	0.884	0.915	0.926	0.963	1	0.954	0.57	0.86	0.054	0.027	0.115	0.165	-0.198	0.051	0.01
HLADRA	0.687	0.882	0.762	0.962	0.12	0.665	0.921	0.988	0.973	0.896	0.966	0.92	0.954	1	0.667	0.878	0.033	0.051	0.145	0.224	-0.138	0.116	-0.025
PDL1	0.572	0.748	0.673	0.701	0.349	0.562	0.561	0.659	0.697	0.567	0.627	0.625	0.57	0.667	1	0.732	-0.092	0.144	0.403	0.489	-0.084	-0.127	-0.099
PDL2	0.51	0.752	0.593	0.887	0.119	0.447	0.759	0.875	0.814	0.918	0.787	0.812	0.86	0.878	0.732	1	-0.015	0.139	0.2	0.308	-0.146	-0.125	-0.067
B7H3	0.284	0.015	0.032	-0.026	0.024	0.283	0.289	0.018	0.053	-0.035	0.123	0.101	0.054	0.033	-0.092	-0.015	1	-0.334	0.073	-0.563	-0.354	0.42	0.167
TNFRSF6B	0.1	0.262	0.232	0.211	0.114	0.124	-0.068	0.045	0.027	0.121	-0.003	-0.03	0.027	0.051	0.144	0.139	-0.334	1	0.569	0.25	0.028	-0.184	-0.185
GAL9	0.317	0.411	0.396	0.24	0.452	0.424	0.19	0.123	0.142	0.083	0.175	0.174	0.115	0.145	0.403	0.2	0.073	0.569	1	0.021	-0.107	-0.02	-0.046
CD47	-0.041	0.246	0.208	0.302	-0.06	-0.041	0.004	0.227	0.234	0.29	0.112	0.102	0.165	0.224	0.489	0.308	-0.563	0.25	0.021	1	0.144	-0.353	-0.184
CD200	-0.156	-0.119	0.001	-0.051	0.065	-0.157	-0.267	-0.13	-0.112	-0.129	-0.174	-0.218	-0.198	-0.138	-0.084	-0.146	-0.354	0.028	-0.107	0.144	1	-0.116	0.266
HVEM	0.368	0.133	0.233	0.044	0.019	0.375	0.262	0.084	0.168	-0.099	0.218	0.085	0.051	0.116	-0.127	-0.125	0.42	-0.184	-0.02	-0.353	-0.116	1	-0.063
CD155	-0.099	-0.058	-0.099	-0.005	0.3	-0.068	0.006	-0.028	-0.074	0.022	-0.066	0.025	0.01	-0.025	-0.099	-0.067	0.167	-0.185	-0.046	-0.184	0.266	-0.063	1

**Fig 4:**
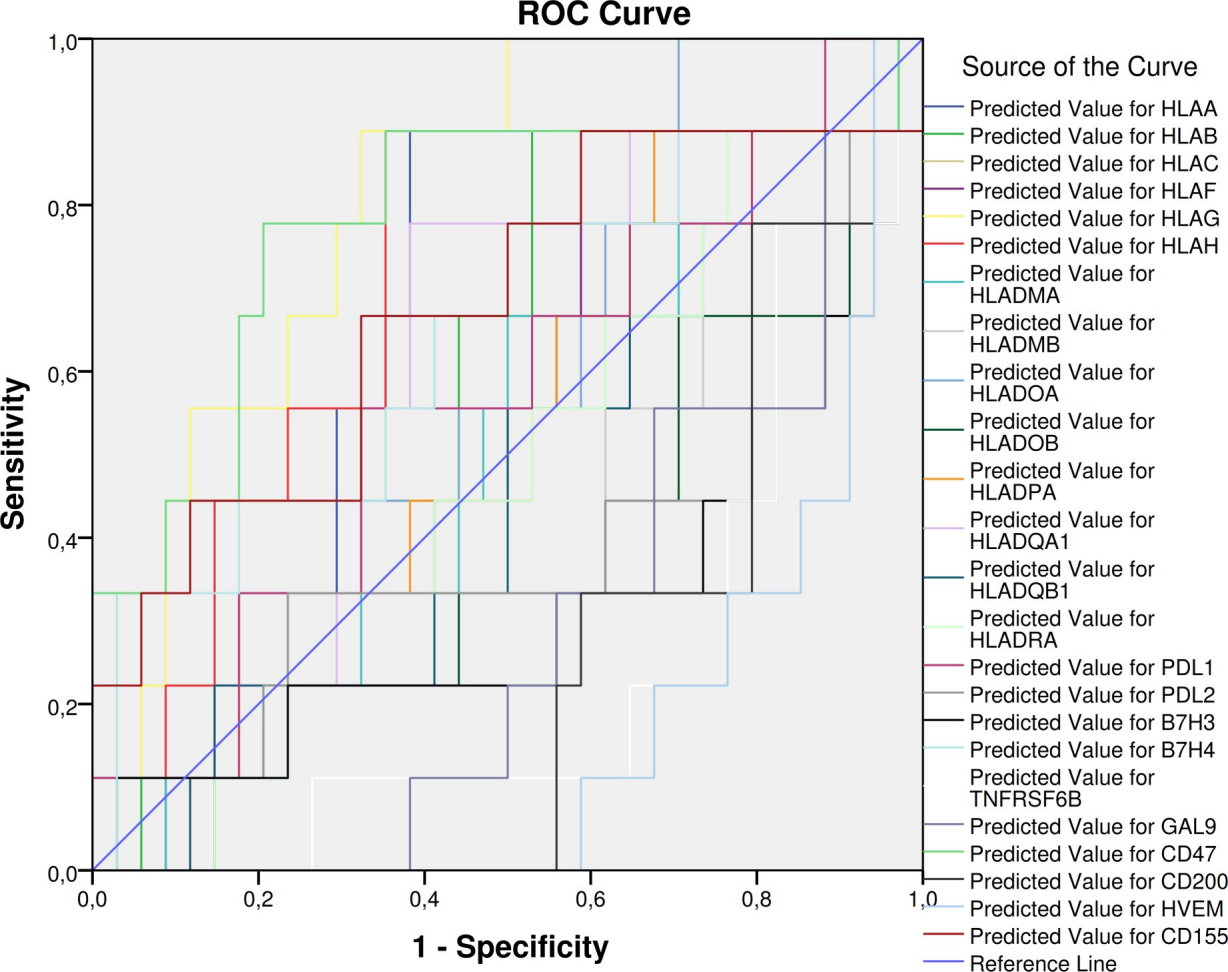
Receiver Operating Characteristic (ROC) curves were constructed for the genes of interest using disease status (recurrent disease) as nominal variable. Predicted values of the genes of interest were generated using General Linear Model to account for clinical confounding variables.

**Fig 5:**
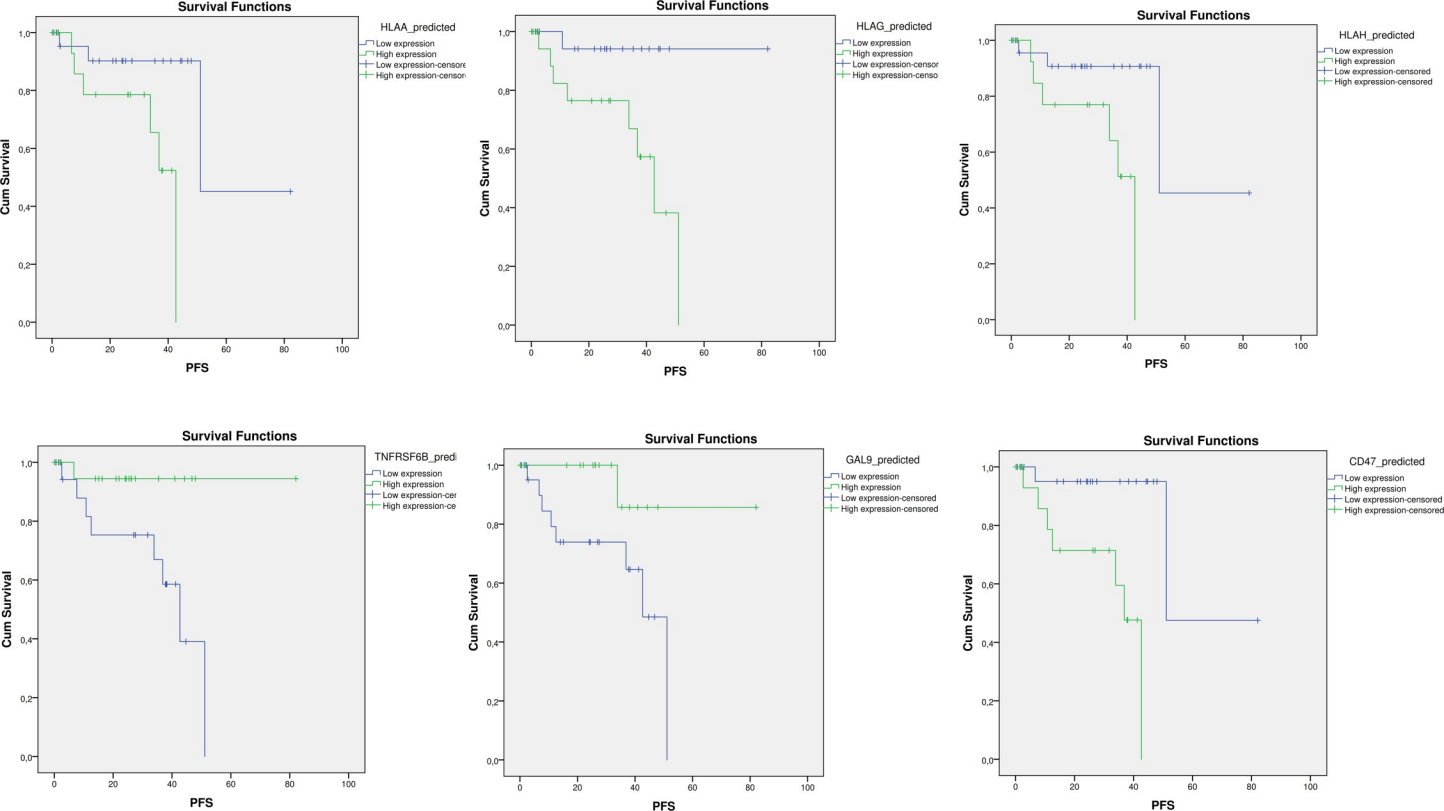
For Kaplan-Meier analysis, predicted values of the genes of interest were generated using General Linear Model to account for clinical confounding variables and were dichotomized using median value as cut-off. For each gene, Log-rank (Mantel-Cox) analysis was performed to assess statistical differences in Progression-Free Survival time between patients with Low and High expression. Only graphs for significant data are shown.

Receiver Operating Characteristic (ROC) curves were constructed using disease status (recurrent disease) as nominal variable. HLA-G resulted to be a significant predictor of recurrent disease, with an AUC of 0.810 (p = 0.05) (Fig 4; Table 3). Among the immune-checkpoint genes, the best predictors found were CD47, with an AUC of 0.781 (p = 0.01); TNFRSF6B, with an AUC of 0.235 (p = 0.016); CD200 (AUC = 0.242; p = 0.018); HVEM (AUC = 0.163; p = 0.002); GAL9 (AUC = 0.284; p = 0.049)(Fig 4; Table 3). Accordingly to the ROC analysis, Kaplan-Meyer analysis revealed that higher levels of the MCH class I molecules—HLA-A, HLA-G, HLA-H—were associated to significantly lower disease-free survival time (Table 4; Fig 5). Among the immune checkpoints, low levels of CD47 expression resulted in higher disease free survival, while higher expression of TNFRSF6B and GAL9 resulted in longer disease free survival (Table 4; Fig 5).

**Table 3:** Data from Receiver Operating Characteristic (ROC) curves generated for the genes of interest using disease status (recurrent disease) as nominal variable. Predicted values of the genes of interest were generated using General Linear Model to account for clinical confounding variables.

Area Under the Curve					
Test Result Variable(s)	Area	Std. Error	Asymptotic Sig.	Asymptotic 95% Confidence Interval	
				Lower Bound	Upper Bound
Predicted Value for HLAA	.667	.101	.128	.468	.865
Predicted Value for HLAB	.585	.101	.438	.386	.784
Predicted Value for HLAC	.542	.107	.698	.332	.753
Predicted Value for HLAF	.533	.105	.765	.327	.739
Predicted Value for HLAG	.810	.069	**.005**	.674	.947
Predicted Value for HLAH	.706	.102	.060	.506	.906
Predicted Value for HLADMA	.497	.105	.976	.292	.702
Predicted Value for HLADMB	.408	.114	.403	.185	.632
Predicted Value for HLADOA	.575	.098	.492	.382	.768
Predicted Value for HLADOB	.369	.109	.232	.156	.582
Predicted Value for HLADPA	.523	.104	.834	.319	.727
Predicted Value for HLADQA1	.598	.104	.370	.395	.801
Predicted Value for HLADQB1	.454	.108	.676	.243	.665
Predicted Value for HLADRA	.487	.106	.905	.280	.694
Predicted Value for PDL1	.572	.108	.511	.360	.784
Predicted Value for PDL2	.382	.122	.282	.144	.621
Predicted Value for B7H3	.320	.114	.101	.096	.545
Predicted Value for B7H4	.644	.113	.189	.423	.865
Predicted Value for TNFRSF6B	.235	.084	**.016**	.070	.401
Predicted Value for GAL9	.284	.085	**.049**	.117	.452
Predicted Value for CD47	.781	.102	**.010**	.580	.982
Predicted Value for CD200	.242	.076	**.018**	.094	.390
Predicted Value for HVEM	.163	.063	**.002**	.040	.287
Predicted Value for CD155	.676	.112	.107	.457	.896

**Table 4:** For Kaplan-Meier analysis, predicted values of the genes of interest were generated using General Linear Model to account for clinical confounding variables and were dichotomized using median value as cut-off. For each gene, Log-rank (Mantel-Cox) analysis was performed to assess statistical differences in Progression-Free Survival time between patients with Low and High expression.

		Estimate	Std. Error	95% Confidence Interval		Log Rank (Mantel-Cox)	
				Lower Bound	Upper Bound	Chi-Square	Sig.
HLAA_predicted	Low expression	60.849	10.617	40.040	81.658	5.316044951	**0.021129918**
	High expression	33.412	4.032	25.509	41.315		
HLAB_predicted	Low expression	55.946	10.201	35.952	75.939	0.869944517	0.350970669
	High expression	37.788	3.113	31.685	43.890		
HLAC_predicted	Low expression	55.442	10.217	35.417	75.468	0.651291391	0.419651352
	High expression	37.976	2.947	32.201	43.751		
HLAF_predicted	Low expression	53.142	9.316	34.883	71.400	0.171876007	0.678449895
	High expression	36.567	2.563	31.544	41.589		
HLAG_predicted	Low expression	77.906	4.068	69.933	85.880	5.92839856	**0.014898775**
	High expression	36.197	4.697	26.991	45.403		
HLAH_predicted	Low expression	61.117	10.602	40.337	81.897	5.989849457	**0.01438843**
	High expression	32.887	4.265	24.528	41.246		
HLADMA_predicted	Low expression	52.667	9.338	34.365	70.970	0.082972562	0.773308785
	High expression	36.747	2.424	31.996	41.498		
HLADMB_predicted	Low expression	52.146	9.366	33.789	70.503	0.002775545	0.957984135
	High expression	37.265	2.248	32.858	41.672		
HLADOA_predicted	Low expression	55.442	10.217	35.417	75.468	0.651291391	0.419651352
	High expression	37.976	2.947	32.201	43.751		
HLADOB_predicted	Low expression	50.167	9.126	32.281	68.054	0.389689022	0.53246291
	high expression	39.300	1.184	36.979	41.621		
HLADPA_predicted	Low expression	54.889	10.239	34.820	74.958	0.470277913	0.492859343
	High expression	38.138	2.806	32.638	43.638		
HLADQA1_predicted	Low expression	57.875	10.408	37.476	78.274	2.255889815	0.133106926
	high expression	35.569	3.732	28.254	42.883		
HLADQB1_predicted	Low expression	52.947	9.253	34.811	71.082	0.132323225	0.716035297
	High expression	36.112	2.963	30.304	41.920		
HLADRA_predicted	Low expression	54.889	10.239	34.820	74.958	0.470277913	0.492859343
	High expression	38.138	2.806	32.638	43.638		
PDL1_predicted	Low expression	58.256	10.422	37.829	78.684	2.587052616	0.107740658
	High expression	35.469	3.734	28.150	42.788		
PDL2_predicted	Low expression	53.142	9.316	34.883	71.400	0.171876007	0.678449895
	High expression	36.567	2.563	31.544	41.589		
B7H3_predicted	Low expression	47.795	9.010	30.135	65.454	1.235888265	0.266264959
	High expression	43.523	1.969	39.663	47.383		
TNFRSF6B_predicted	Low expression	36.302	4.726	27.039	45.564	5.737360787	**0.016607729**
	High expression	77.909	4.072	69.927	85.892		
GAL9_predicted	Low expression	37.204	4.571	28.244	46.164	4.368411636	**0.036611103**
	High expression	75.214	6.375	62.719	87.709		
CD47_predicted	Low expression	63.613	10.808	42.430	84.796	9.483907795	**0.00207282**
	High expression	31.139	4.341	22.630	39.647		
HVEM_predicted	Low expression	46.037	8.365	29.642	62.431	2.86334347	0.090618963
	High expression	45.926	1.856	42.289	49.563		
CD155_predicted	Low expression	46.192	4.012	38.328	54.056	1.343753464	0.24637312
	High expression	47.095	10.294	26.920	67.271		

## Discussion

Metastatic UM represents an unmet medical need characterized by extremely poor prognosis [1,2]. Recently, gene expression and genomic analysis have identified different subsets of uveal cancer that could propel future efforts to design more individualized and effective treatment strategies [16]. The immune-based therapeutic strategies, that have increased the overall survival of cutaneous melanoma patients, have not yet led to significant improvement in unresectable/metastatic UM patients [17]. Nevertheless, the growing knowledge of cancer immunology suggests the possibility of novel therapeutic strategies.

In the present study, we wanted to formally characterize the transcriptomic features regulating the immunogenicity of UM and to determine whether T cell-mediated inflammation is able to affect the expression profile of genes putatively involved in immune-escape. In comparison to normal uveal melanocytes, significant lower levels of MHC genes were found in UM cells. Also, most of the inhibitory immune-checkpoints analyzed were not different in uveal cancer, with the exception of CD200 and HVEM, that resulted significantly reduced. Interestingly, the expression levels of several MHC class I and II molecules, as well as of PDL1 and PDL2 significantly increased under inflammatory conditions. It is reasonable that IFN-gamma may partly be responsible for the upregulation of these molecules, as in vitro treatment of the UM cell line, 92.1, with IFN-gamma, was associated to a significant increase in the expression of these genes (S1 Fig). Also, these observations are in line with previous reports by independent Authors who showed that IFN-gamma stimulation is able to affect the expression the localization of these molecule in UM, in turn resulting in an altered immune response of effector cells [9,18,19].

It is worth pointing out that our analysis of the TGCA data have been performed accounting for the presence of BAP1 mutation as confounding variable. Robertson et al. [20] have reported that UM with poor prognosis initially develops monosomy 3 (M3-UM), followed by BAP1 alterations that are associated with a unique global DNA methylation profile. They reported 4 transcription-based UM subsets, in which one cluster corresponded to M3-UM with immune infiltration. Also, They showed that a CD8 T cell infiltrate was present in 30% of M3-UM, while nearly absent in D3-UM, and interferon-gamma signaling (IFNG, IFNGR1, and IRF1), T cell invasion (CXCL9 and CXCL13), cytotoxicity (PRF1 and GZMA), and immunosuppression (IDO1, TIGIT, IL6, IL10, and FOXP3) were co-expressed with CD8a, suggesting that promotion of an immune environment plays a significant role in aggressive UM, i.e., M3 UM. Notably, no significant differences could be found in the expression of the genes here analyzed between BAP1 mutated and wild type samples (data not shown). In addition, we have provided data on the expression of the genes analyzed in our paper after stable loss of BAP1 in three UM cell lines. As it is shown in S2 Fig, the genes analyzed are not significantly modulated in the BAP1 deficient cell lines, with the exception of PDL-1 and HLA-A with resulted downregulated upon BAP1 loss (p = 0.022 and p = 0.031, respectively). Although these data may apparently contradict the negative prognostic value of BAP1 loss/mutation, however, they further support the important role of additional factors in the pathophysiological mechanisms underlying tumor growth, immune escape and prognosis in UM.

PDL1 inversely correlated with OS, PFS and thickness of the tumor. These findings have important clinical implications for the role of immunotherapies in the treatment of patients with UM. Unfortunately, to date, disappointing results have been obtained from Phase II clinical trials on unresectable/metastatic UM patients treated with the monoclonal antibodies targeting the PD1/PDL1 axis, pembrolizumab, nivolumab or atezolizumab [21]. These results are generally ascribed to the lack of expression of PDL1 in UM, as determined by independent Authors. Indeed, Javed et al. [22] have shown that only 5.1% of metastatic UM specimens expressed PDL1, while Kaunitz et al. [23] found that approximately 10% of UM expressed PDL1 and only correlated with a moderate–severe grade of CD8+ TILs. The data obtained from the TGCA database here analyzed confirm the low expression of PDL1, as the mean mRNA copy number was scarce (mean = 19.2; data not shown). However, the inverse correlation with the clinical data of the patients seem to point to an important role of this gene in UM. Additional markers of tumor immunogenicity and/or immune checkpoint molecules are needed, which may fine-tune the effector activity of tumor-infiltrating T cells and influence patients’ prognosis. In fact, it is reasonable that functional redundancy occurs between alternative immune-inhibitory pathways and PDL1, so that combinatorial therapies may reshape the balance between activation and inhibitory signals received by immune infiltrating cells, thus restoring effective antitumor T-cell immunity.

It is also notable the inverse correlation between HLA Class 1 genes expression and patients’ survival. This particular data fits in with the trend of increase in MHC expression that we observed under inflammatory conditions. Our data are in line with van Essen and collaborators (2016) showing that increased HLA class I levels are positively associated to a high infiltrate of CD3+, and CD68+ leukocytes. Accordingly, these Authors found that UM cells grafted in SCID-mice (lacking T and B cells) express the HLA class I and II genes at a much lower extent than their original counterpart [24]. Moreover, lack of HLA class I expression correlates with higher OS [25]. It is likely that this strategy may allow the tumor to escape the immune surveillance by NK cells [25], while the increase in PDL1 may block CTL response [25].

From the ROC and Kaplan-Meier analysis, a potential prognostic value for GAL9 and TNFRSF6B was also determined. However, the role of these genes in UM is still elusive. In fact, despite their immune-suppressive properties, we found that higher levels of expression are associated to better PFS. GAL9 encodes for a protein with affinity to bind β-galactoside and that possess carbohydrate recognition domains [26]. It was previously shown that GAL9, via interacting with TIM-3, is able to negatively regulate Th1 responses, to induce apoptosis of CTLs and to promote suppressive activity of Tregs. On the other hand, in a preclinical model of melanoma, GAL9 was found to expand plasmacytoid cell-like macrophages, thus increasing the NK-mediated cytolysis of cancer cells. Therefore, GAL9 may exert pleiotropic effects in cancer and the prognostic value of this gene in cancer remains controversial. In fact, a meta-analysis of fourteen studies, involving 2326 solid cancer patients, has recently revealed that high GAL9 expression was associated to improved overall survival, and was significantly correlated with smaller depth of invasion, earlier histopathological stage, negative lymph node metastasis and negative distal tumor metastasis [26].

TNFRSF6B is a member of the tumor necrosis factor receptor superfamily that acts as a decoy receptor for the apoptosis-inducing proteins Fas ligand (FasL), lymphotoxin analogues (LIGHT), and tumor necrosis factor-like ligand 1A (TL1A). In solid tumors, TNFRSF6B overexpression has been significantly associated with worse OS in patients with solid tumors, but its expression does not influence recurrence-free survival [27]. Additional functional data are needed to decipher the exact role of TNFRSF6B in regulating cancer recurrence, immune response to cancer and patients’ survival in UM.

Finally, for the first time, we propose a possible role for CD47 in the immune evasive properties of UM. CD47 is a molecule with an immunoglobulin-like domain that is expressed on tumor cell surface and inhibits macrophage phagocytosis via binding the signal regulatory protein α (SIRPα) on phagocytes [28]. In gastric cancer, CD47 expression is an independent negative prognostic factor for gastric cancer, and in ovarian cancer, it is associated with adverse clinical characteristics and a poor prognosis [28]. We show here that CD47 is significantly upregulated by UM cells following inflammatory stimuli and that it represents a good independent predictor of disease progression. Anti-CD47-targeted therapy may represent a promising strategy to treat cancer and to improve long-term survival. A number of potent CD47 inhibitors are currently available (e.g. Hu5F9-G4, CC-90002, TTI-621, NI-1701, NI-1801, SRF231) and some of them are already under clinical investigation in human solid and hematological cancers [28].

Our *in silico* analysis highlights the complex pattern of gene expression that may orchestrate the interaction between UM cells and the immune cells and warrants further investigation aimed at exploring more focused pathogenic-tailored immunotherapeutic approaches in patients with UM.

## Supporting information

S1 Fig IFN-gamma drives immune escape in uveal melanoma. The uveal melanoma 92.1 cells were exposed for 72 hrs to recIFN-gamma 200 ng/ml and the expression levels of the HLA-A, HLA-DOB, PDL-1 PDL-2 and CD47 genes were determined by Real-time PCR. Primer pairs sequences were obtained from the PrimerBank database. The data are presented as fold change increase with respect to unstimulated control cells and CI 95%. Statistical differences have been determined by two-tailed unparired Student’s t test using the GrapPad Prism software.
(TIF)

S2 Fig BAP1 loss does not affect the expression of immune evading genes in uveal melanoma. To determine whether stable loss of the BAP1 gene altered the immunological profiling of uveal melanoma, the GSE48863 microarray dataset was analyzed. GSE48863 included data from the three human uveal melanoma cell lines, 92.1, Mel290, and OCM1a, that were stably depleted using shRNA for either BAP1 or the control gene GFP. Cells were grown under puromycin selection for 4 weeks after lentiviral infection and microarray was performed using the Illumina HumanHT-12 V4.0 expression beadchip platform. Two biological replicates were performed for each cell line. An heatmap showing the expression levels of the genes of interest as fold change with respect to the relative control cells is presented. The heatmap is color coded from blue to red and the level of expression of each gene in the control cells is arbitrarily set to 1.
(TIF)
