# Supplementary figures and images for: Differential modulation and prognostic values of immune-escape genes in uveal melanoma
Source: PLoS One. 2019 Jan 17;14(1):e0210276. doi: 10.1371/journal.pone.0210276 (PMC6336329; doi:10.1371/journal.pone.0210276)

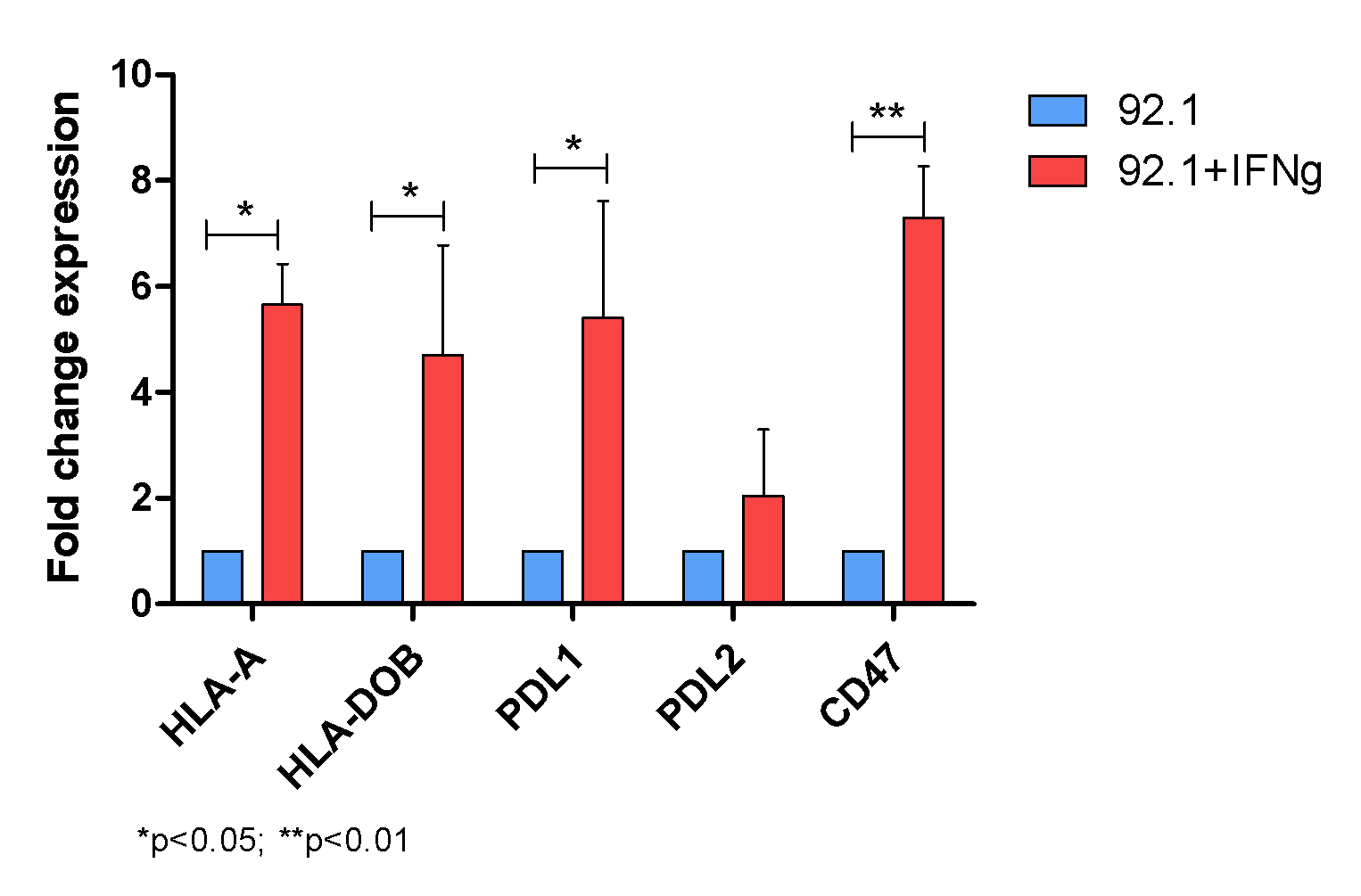

Supplement: S1 Fig — The uveal melanoma 92.1 cells were exposed for 72 hrs to recIFN-gamma 200 ng/ml and the expression levels of the HLA-A, HLA-DOB, PDL-1 PDL-2 and CD47 genes were determined by Real-time PCR. Primer pairs sequences were obtained from the PrimerBank database. The data are presented as fold change increase with respect to unstimulated control cells and CI 95%. Statistical differences have been determined by two-tailed unparired Student’s t test using the GrapPad Prism software. (TIF) [file pone.0210276.s001.tif]

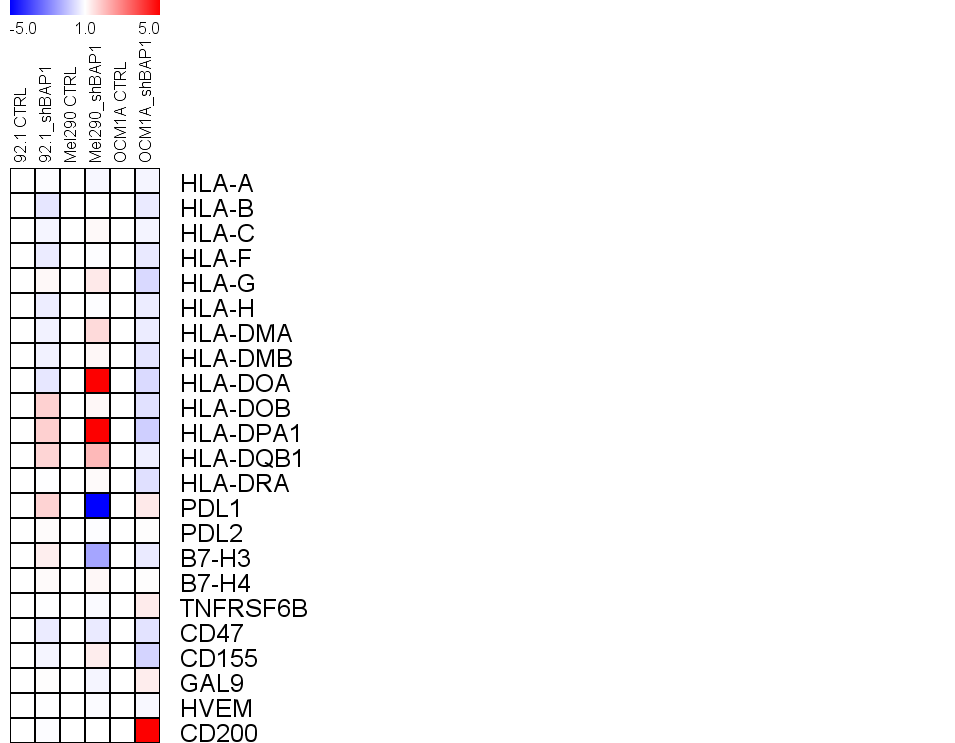

Supplement: S2 Fig — To determine whether stable loss of the BAP1 gene altered the immunological profiling of uveal melanoma, the GSE48863 microarray dataset was analyzed. GSE48863 included data from the three human uveal melanoma cell lines, 92.1, Mel290, and OCM1a, that were stably depleted using shRNA for either BAP1 or the control gene GFP. Cells were grown under puromycin selection for 4 weeks after lentiviral infection and microarray was performed using the Illumina HumanHT-12 V4.0 expression beadchip platform. Two biological replicates were performed for each cell line. An heatmap showing the expression levels of the genes of interest as fold change with respect to the relative control cells is presented. The heatmap is color coded from blue to red and the level of expression of each gene in the control cells is arbitrarily set to 1. (TIF) [file pone.0210276.s002.tif]
